# Melanin-Based Coatings as Lead-Binding Agents

**DOI:** 10.1155/2012/361803

**Published:** 2012-04-26

**Authors:** Karin Sono, Diane Lye, Christine A. Moore, W. Christopher Boyd, Thomas A. Gorlin, Jason M. Belitsky

**Affiliations:** Department of Chemistry and Biochemistry, Oberlin College, 119 Woodland Street, Oberlin, OH 44074, USA

## Abstract

Interactions between metal ions and different forms of melanin play significant roles in melanin biochemistry. The binding properties of natural melanin and related synthetic materials can be exploited for nonbiological applications, potentially including water purification. A method for investigating metal ion-melanin interactions on solid support is described, with lead as the initial target. 2.5 cm discs of the hydrophobic polymer PVDF were coated with synthetic eumelanin from the tyrosinase-catalyzed polymerization of L-dopa, and with melanin extracted from human hair. Lead (Pb^2+^) binding was quantified by atomic absorption spectroscopy (flame mode), and the data was well fit by the Langmuir model. Langmuir affinities ranged from 3.4 · 10^3^ to 2.2 · 10^4^ M^−1^. At the maximum capacity observed, the synthetic eumelanin coating bound ~9% of its mass in lead. Binding of copper (Cu^2+^), zinc (Zn^2+^), and cadmium (Cd^2+^) to the synthetic-eumelanin-coated discs was also investigated. Under the conditions tested, the Langmuir affinities for Zn^2+^, Cd^2+^, and Cu^2+^ were 35%, 53%, and 77%, respectively, of the Langmuir affinity for Pb^2+^. The synthetic-eumelanin-coated discs have a slightly higher capacity for Cu^2+^ on a per mole basis than for Pb^2+^, and lower capacities for Cd^2+^ and Zn^2+^. The system described can be used to address biological questions and potentially be applied toward melanin-based water purification.

## 1. Introduction

While the term “melanin” is widely recognized by the general public, our understanding of the bioorganic chemistry of melanins is far less advanced than for other biopolymers. However, there have been recent advances in the understanding of melanin structure, function, and properties [1, 2], as well as in the development of well-defined synthetic mimics [3, 4] and melanin-based materials [5, 6]. Melanins display extensive molecular recognition chemistry, with the ability to bind a wide range of organic and inorganic species. The interaction of melanins with metal ions plays a particularly large role in their biochemistry [7]. Current research suggests that the black-to-brown pigment eumelanin is formed via supramolecular assembly, and metal ions may be integral components of this assembly process [7, 8]. In human melanocytes, eumelanin is produced along with pheomelanin, a chemically distinct yellow-to-red pigment, in organelles known as melanosomes. Melanosomes are thought to play a role in calcium homeostasis [9]. Interactions between metals and melanin have been proposed to play a role in the development of melanoma [10] and may be leveraged for potential treatment [11]. In the substancia nigra region of the brain, the redox balance between iron, dopamine, neuromelanin, and sources of oxidative stress is thought to play a role in Parkinson's disease [12]. Recently, a new form of neuromelanin found widely throughout the brain, nanoparticle aggregates with a eumelanin-like exterior, was shown to be a protective reservoir for heavy metals, including lead [13]. The sequestration of metal ions by melanin has been exploited in forensics and toxicology [14], and most recently, paleontology [15]. Interactions between melanins and metal ions can also be leveraged for nonbiological applications [16, 17], potentially including water purification [18, 19]. 

**Figure 1:**
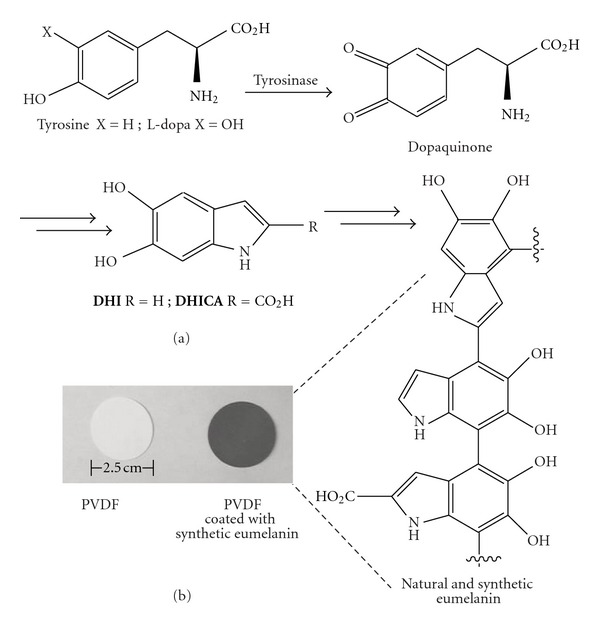
(a) Biosynthesis of natural eumelanin and biomimetic production of synthetic eumelanin. (b) 2.5 cm PVDF disc before (left) and after (right) coating with synthetic eumelanin.

Eumelanin biosynthesis is initiated by the enzyme tyrosinase, which carries out two successive oxidations of tyrosine, first to L-dopa and then to dopaquinone (Figure 1(a)). Intramolecular ring closure ultimately leads, with and without loss of the carboxylic acid, to the eumelanin monomers 5,6-dihydroxyindole (DHI) and 5,6-dihydroxyindole-carboxylic acid (DHICA), respectively. These are further oxidized to highly reactive indolequinones, which react with each other and other components of the melanosome to form eumelanin. DHI and DHICA are now thought to form relatively short covalent oligomers that are heterogeneous with respect to both linkages between dihydroxyindole units and their oxidation states. These heterogeneous oligomers aggregate to form nanoparticles, and eumelanin superstructures are formed from these nanoparticles [1–4, 8]. Along with hydrogen bonding and aromatic stacking, metal ions (particularly Fe^3+^, Cu^2+^, Zn^2+^, Mg^2+^, and Ca^2+^) are thought to provide key bridging interactions between both oligomers and nanoparticles and may also act as templates for different oligomeric and supramolecular structures [7, 8]. Changes in the aggregation and nanoscale morphology of preformed synthetic eumelanin have been observed upon the addition of Cu^2+^ and K^+^ [8, 20]. Endogenously bound metal ions can be removed from natural samples such as *Sepia *eumelanin from cuttlefish ink and rebound with high affinity [21]. 

*In vitro*, under ambient oxygen conditions, conversion of L-dopa to a DHI/DHICA-containing synthetic eumelanin material (Figure 1) is thermodynamically spontaneous and kinetically facile from the dopaquinone oxidation state [22]. Thus, numerous methods can be used to initiate the formation of synthetic eumelanin from L-dopa, including autooxidation, chemical oxidants, and biomimetic catalysis with tyrosinase [22]. The quinone intermediates in these polymerizations are sufficiently reactive that many other compounds can potentially be incorporated covalently into the resulting materials, potentially tuning their properties. (Nicotine is a physiologically relevant example [23].) Additionally, Messersmith, Ball, and others have found that eumelanin-related polydopamine coatings can adhere to nearly any surface [5, 24]. Thus, synthetic eumelanin can be readily prepared, modified, and presented in a variety of formats. Whereas synthetic chemistry offers the potential for greater variation, the ability to make use of melanin from renewable natural resources is attractive from a sustainability perspective. Wherever there are people there is hair, such that melanin from human hair [25–27] could be useful for water purification in areas where there are few other resources.

 As part of a broader program to understand and modulate [28] eumelanin self-assembly and subsequent molecular recognition, we have developed a convenient method for studying metal ion binding to both synthetic and naturally derived melanin-based materials on solid support. Lead was chosen as initial target due to its toxicity, environmental distribution, and accumulation in neuromelanin [13]. There is great interest in the development of heavy metal sequestration agents based on synthetic systems [29, 30], as well as natural, renewable, and/or otherwise-discarded materials [31, 32]. Two recent studies have investigated melanin from squid ink for binding lead (Pb^2+^) and cadmium (Cd^2+^) [18] and fungal melanin secretions for binding chromium (Cr^6+^) [19]. For water purification applications under batch conditions, an advantage of the presentation of melanin as a coating on solid support compared to a free suspension is that the solid support obviates the need for filtration or sedimentation steps to remove the suspension. As a research method, the advantages of coatings rather than free suspensions include ease of handling, minimization of the volume of heavy metal waste, and potential for combinatorial variation of synthetic coatings. Importantly, the solid support chosen, polyvinylidene di-fluoride (PVDF), is amenable to coating with both natural and synthetic melanin samples, allowing their direct comparison under identical conditions. Overall the process described here is complementary to other recent approaches for investigating melanin-metal ion interactions [7, 8, 33]. Here we introduce a metal binding assay using PVDF discs coated with synthetic eumelanin and melanin extracted from human hair, with a focus on lead binding.

## 2. Experimental

### 2.1. Synthetic-Eumelanin-Coated Discs

The production of synthetic eumelanin via the biomimetic oxidation of L-dopa with tyrosinase follows standard practice [22, 23, 34–36]. The discs are prepared in batches of twelve. Polyvinylidene di-fluoride (PVDF) discs are prepared from BioTrace PVDF membrane sheets (Pall) using a 2.5 cm diameter arch punch. 2.5 cm PVDF discs are shaken in MeOH for ~1 min, followed by two water washes (6 discs per 50 mL H_2_O, 5 min each). A mushroom tyrosinase (Aldrich) stock solution of 1000 units/mL in sodium phosphate buffer (50 mM, pH 7.0) is prepared. L-dopa is dissolved in the same buffer to a concentration of 5.08 mM (1 mg/mL). 84 mL of freshly prepared L-dopa solution is added to a wide-mouth glass jar followed by 850 *μ*L of the tyrosinase stock solution. Twelve PDVF discs are added to this solution (5.02 mM L-dopa, 10 units/mL tyrosinase, 50 mM sodium phosphate, pH 7.0), and the jar is shaken at 250 rpm for ~22 hrs at room temperature. After shaking for ~22 hrs, the discs are coated synthetic eumelanin and black in appearance. They are washed twice in the sodium phosphate buffer (6 discs per 50 mL buffer, 5 min each). Synthetic-eumelanin-coated discs are stored immersed in sodium phosphate buffer (50 mM, pH 7.0), in the dark at room temperature.

 To determine the amount of synthetic eumelanin per disc, ≥10 discs from different polymerizations were stored in a dessicator until dryness and compared to blank PVDF discs treated in the same manner. Approximately 1.5 mg are added per disc as a result of the biomimetic polymerization (average mass of “blank” PDVF disc 36.87 ± 0.38 mg, average mass of synthetic-eumelanin-coated disc 38.40 ± 0.40 mg).

### 2.2. Hair-Extract-Coated Discs

The extraction of melanin from human hair via an acid-base method [26, 27], described in detail below, follows the procedure of Haywood et al. with a wash/precipitation sequence modified from Liu et al. to include a step of removal of endogenous metal ions. The presence of extracted melanin throughout the procedure and its reduction following deposition on PVDF was verified by spectroscopic comparison with synthetic eumelanin standards [25, 26].

 Five grams of human hair produce 40 coated discs by the following procedure. Black (Indian) hair (5 g) cut in ~5 mm pieces is added to 250 mL 1 M NaOH (aq.) in a 1 L beaker at 90°C, and the mixture is stirred for 10 minutes at 90°C. The beaker is cooled on ice, and the total volume is returned to 250 mL by adding water. The mixture is centrifuged at 650 g for 5 min. The supernatant is transferred to new centrifuge vessels and adjusted to pH ≤ 3 by addition of con. HCl, inducing precipitation. The precipitate is collected by centrifugation at 14680 g for 10 min. The supernatant is discarded and the precipitate is resuspended in 200 mL sodium phosphate buffer (50 mM, pH 4.50). The pH of this suspension is adjusted to between pH 4.5 and pH 5.0 by addition of 3 M NaOH. The suspension is mixed thoroughly, then centrifuged at 14680 g for 10 min. The supernatant is discarded and the precipitate is resuspended in 200 mL sodium ethylenediaminetetraacetic acid buffer (Na_2_EDTA, 100 mM, pH 4.50). After thorough mixing, the suspension is centrifuged at 14680 g for 10 min, and then the supernatant is discarded. The precipitate is resuspended in 200 mL sodium phosphate buffer (50 mM, pH 4.50), mixed thoroughly, and then the suspension is centrifuged at 14680 g for 10 min. After discarding the supernatant, the precipitate is resuspended in 152 mL sodium phosphate buffer (50 mM, pH 4.50) and mixed thoroughly. Forty 2.5 cm PVDF discs are shaken in MeOH for ~1 min, followed by two water washes (up to 6 discs per 50 mL H_2_O, 5 min each). The 152 mL melanin suspension in pH 4.50 phosphate buffer is adjusted to pH 2.20 (±0.05) and distributed to 4 wide-mouth glass jars, 38 mL per jar. 12.667 mL of formamide (to 25% v/v, 50.667 mL total volume) is added to each jar, followed by 10 washed PVDF discs. The jars are shaken at 250 rpm for ~23 hours at room temperature, during which time melanin deposits onto the PVDF discs from the 1 : 3 formamide : phosphate buffer (50 mM, pH 2.20) suspension, forming a macroscopically spotty coating. Hair-extract-coated discs are stored immersed in water, in the dark at room temperature.

 To determine the amount of hair extract per disc, ≥10 discs from different depositions were stored in a dessicator until dryness and compared to blank PVDF discs treated in the same manner. Approximately 3 mg are added per disc as a result of the hair extract deposition (average mass of “blank” PDVF disc 36.87 ± 0.38 mg, average mass of hair-extract-coated disc 40.03 ± 0.52 mg).

### 2.3. Metal Ion-Binding Experiments

Each titration includes 10 concentrations of the nitrate salts of Pb^2+^ (15.1 *μ*M to 2.265 mM), Cu^2+^ (21.5 *μ*M to 3.221 mM), Cd^2+^ (16.2 *μ*M to 3.240 mM), or Zn^2+^ (33.6 *μ*M to 6.720 mM). Pb^2+^ solutions are prepared from Pb(NO_3_)_2_ in pure H_2_O (pH uncorrected) or in 50 mM NaNO_3_ (aq.), with the final pH for each concentration adjusted to pH 4.00, 4.75, or 5.50. Cu^2+^, Cd^2+^, and Zn^2+^ solutions are prepared from Cu(NO_3_)_2_ · 2.5H_2_O, Cd(NO_3_)_2_ · 4H_2_O, and Zn(NO_3_)_2_ · 6H_2_O, respectively, in 50 mM NaNO_3_ (aq.) with the final pH for each concentration adjusted to pH 4.75.

**Figure 2:**
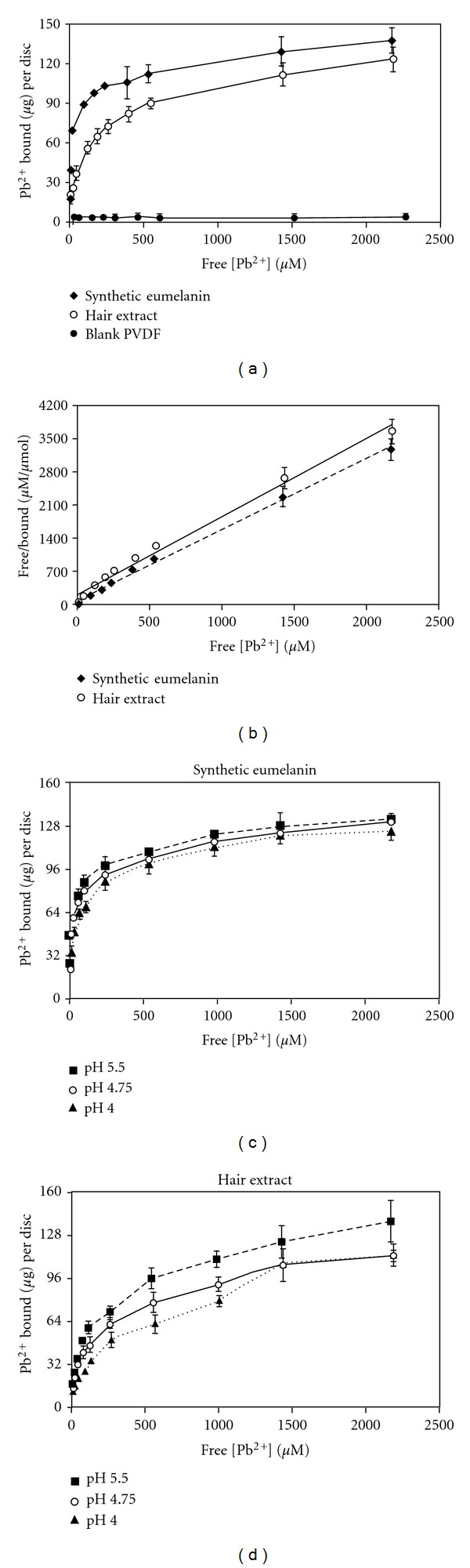
(a) Binding isotherms of Pb^2+^ ions to synthetic-eumelanin-coated discs, hair-extract-coated discs, and blank PVDF discs; pH uncorrected titrations of Pb(NO_3_)_2 _ in H_2_O. (b) Linearized Langmuir isotherm representation of the data for synthetic-eumelanin- and hair-extracted-coated discs in (a), *R* ^2^ ≥ 0.98. (c) Binding isotherms to Pb^2+^ ions to synthetic-eumelanin-coated discs; titrations of Pb(NO_3_)_2 _ in 50 mM NaNO_3 _ (aq.) at initial pH 5.50, 4.75, and 4.00. (d) Binding isotherms to Pb^2+^ ions to hair-extract-coated discs; titrations of Pb(NO_3_)_2 _ in 50 mM NaNO_3 _ (aq.) at initial pH 5.50, 4.75, and 4.00. (a–d) Data are average values from at least three independent titrations, each with different discs; error bars represent one standard deviation.

**Figure 3:**
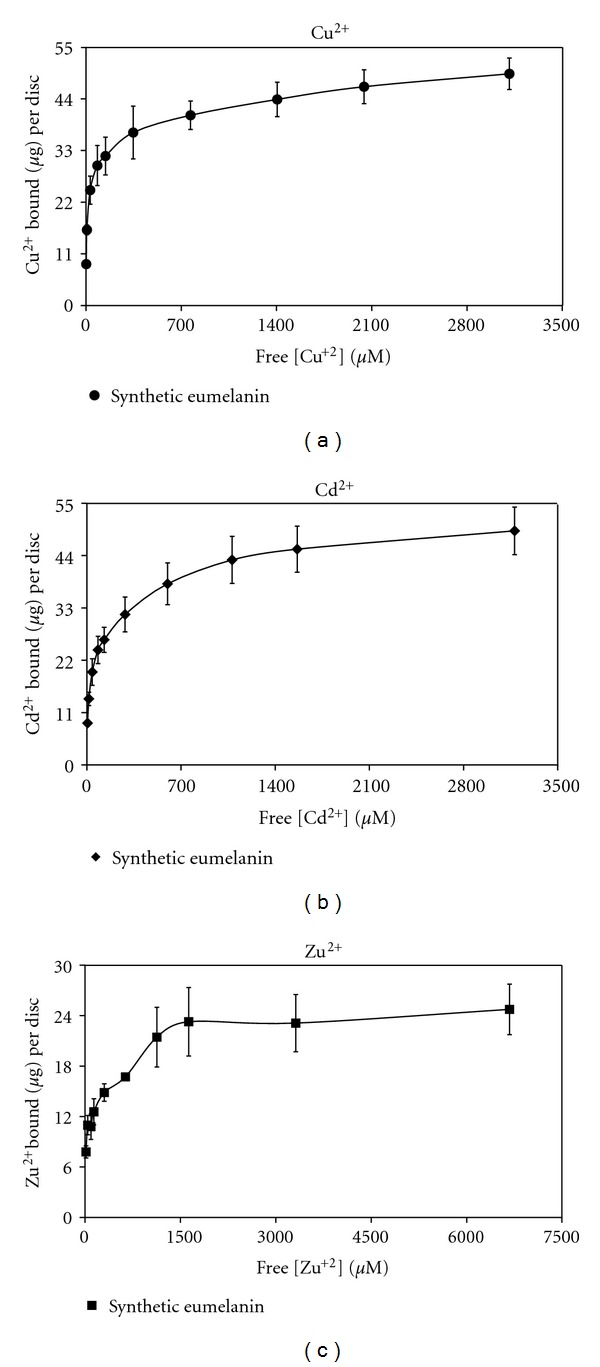
(a) Binding isotherm of Cu^2+^ ions to synthetic-eumelanin-coated discs; titration of Cu(NO_3_)_2_ · 2.5H_2_O in 50 mM NaNO_3 _(aq.) at initial pH 4.75. (b) Binding isotherm of Cd^2+^ ions to synthetic-eumelanin-coated discs; titration of Cd(NO_3_)_2_ · 4H_2_O in 50 mM NaNO_3 _(aq.) at initial pH 4.75. (c) Binding isotherm of Zn^2+^ ions to synthetic-eumelanin-coated discs; titration of Zn(NO_3_)_2_ · 6H_2_O in 50 mM NaNO_3 _(aq.) at initial pH 4.75. (a–c) Data are average values from at least three independent titrations, each with different discs; error bars represent one standard deviation.

 Synthetic-eumelanin- and hair-extract-coated discs are washed twice in water (6 discs per 50 mL H_2_O, 5 min each). Uncoated, blank PVDF discs are shaken in MeOH for 1 min, followed by two water washes (6 discs per 50 mL H_2_O, 5 min each). Using six-well polystyrene plates, a single disc is added to 7 mL per well of a metal ion-containing solution. The six-well plates are covered with tinfoil and shaken at room temperature for ~22 hrs. At the end of this equilibration, discs are removed and washed individually (25 mL H_2_O, 5 min, 2x). A single disc is added to 7 mL of EDTA solution (100 mM Na_2_EDTA, pH 4.50) per well in six-well polystyrene plates. The six-well plates are covered with tinfoil and shaken at room temperature for 30 min. The discs are removed and the EDTA solutions were analyzed using atomic absorption spectroscopy in flame mode. Lead samples were analyzed using a Perkin-Elmer 1100B atomic absorption spectrometer (air-acetylene flame, 0.7 nm slot width, hollow cathode lamp, 283.3 nm). A Perkin-Elmer AAnalyst 700 atomic absorption spectrometer was used to analyze copper (air-acetylene flame, 0.7 nm slot width, hollow cathode lamp, 324.8 nm), cadmium (air-acetylene flame, 0.7 nm slot width, hollow cathode lamp, 228.8 nm), and zinc (air-acetylene flame, 0.7 nm slot width, hollow cathode lamp, 213.9 nm). Metal ion concentrations (in *μ*g/mL) were determined with respect to a standard curve generated during the same session. The total amount of metal ion per EDTA solution (*μ*g/mL × 7 mL volume) is taken to be the total amount (*μ*g) of metal ion bound per disc. The data shown in Figures 2 and 3 and Tables 1, 2, and 3 represent average values from at least three independent titrations, each with different discs. 

## 3. Results and Discussion

Our procedure for investigating melanin-metal ion interactions on solid support begins with the formation of a melanin-based coating on discs of the hydrophobic polymer polyvinylidene di-fluoride (PVDF). The coated discs are then allowed to equilibrate with varying concentrations of the target ion for 22 hours, washed, and immersed in a solution containing the chelating agent EDTA for 30 minutes. The metal ion content of this solution is then analyzed by atomic absorption spectroscopy (flame mode). Preliminary kinetic investigations show that 22 hours is more than sufficient for equilibration. Similarly, 30 minutes in a 100 mM Na_2_EDTA solution (pH 4.50) was sufficient for metal ion extraction, in that further washes did not yield additional removal of metal ions. This does not rule out the possibility of an extremely tight-binding fraction, and weakly bound ions may be lost in the washing steps, such that the procedure may underestimate the total amount of metal ions bound. However, direct measurement of the decrease in metal ion concentration in the equilibration solutions agrees well with the amount recovered in the EDTA washes (data not shown).

Commercially available PVDF membranes (BioTrace, Pall) with 40 micron pores are typically used for bioanalytical applications such as Western blots. PVDF has been previously used as solid-support for metal ion-binding polymer membranes [37] and was chosen for use here because it displays little to no binding to lead ions on its own (Figure 2(a)). Preparation of synthetic eumelanin and deposition onto PVDF was accomplished in a single overnight step. Initiating the biomimetic tyrosinase-catalyzed oxidative polymerization of L-dopa [22] in the presence of 2.5 cm PVDF discs and allowing the reaction to proceed for ~22 hrs at room temperature while shaking at a rate fast enough (250 rpm) to avoid settling or self-aggregation of the hydrophobic discs provides a black synthetic eumelanin coating (Figure 1(b), see Section 2 for detailed procedure). The average mass added per disc during this biomimetic polymerization is on the order of 1.5 mg. Compared to eumelanin samples from natural sources such as *Sepia *ink, synthetic eumelanin materials produced from L-dopa/tyrosinase tend to have a lower DHICA : DHI ratio (i.e., fewer carboxylic acids) and tend to be more amorphous [34].

PVDF discs can also be coated with melanin from human hair (see Section 2 for detailed procedure). Melanin in hair is a mixture of eumelanin and pheomelanin; black hair, used in this study, primarily contains eumelanin [25]. A straightforward acid/base extraction procedure [26, 27] was used, including a short, high temperature, basic heating step (1 M NaOH, 90°C, 10 min), which has been shown to reduce melanin degradation for black hair samples [26] and an EDTA wash to remove endogenously bound metal ions. Following extraction from hair, melanin deposits onto PVDF discs from an acidic 25% formamide solution forming a macroscopically spotty coating. The average mass added per disc as a result of this deposition is on the order of 3 mg, which represents a 2.4% recovery of the mass of hair used. This hair extract coating is likely not completely free of keratin—also a metal-binding agent—and the melanin may experience some degradation, which results in increased carboxylic acid content [27]. Both the hair extract and synthetic eumelanin coatings are stable to shaking and storage in aqueous solutions, without loss of material from the support over the course of shaking for 22 hrs during binding experiments or at least two weeks of storage in water (hair-extract-coated discs) or 50 mM sodium phosphate buffer, pH 7.0 (synthetic-eumelanin-coated discs). If the coated discs are stored at room temperature in aqueous solution on the time-scale of months, some leaching into the solution is observed.

The binding assay outlined above and described in detail in Section 2 was developed with Pb^2+^ as the initial target ion (Figure 2 and Tables 1 and 2). Coated and blank PVDF discs were immersed in aqueous lead nitrate solutions with concentrations ranging from 15.1 *μ*M to 2.265 mM. Following a 22 hr equilibration, the discs were washed in pure water and then exposed to an EDTA solution (100 mM Na_2_EDTA, pH 4.50) for 30 minutes. Lead ions that bound to the discs were extracted into the EDTA solution, which is then quantified by atomic absorption spectroscopy (flame mode, see Section 2). As shown in Figure 2(a), a plot of *μ*g Pb^2+^ bound *per disc *versus free [Pb^2+^], the coated discs show significant binding compared to the negligible results for the blank discs. In our first titrations (Figures 2(a) and 2(b), designated “pH uncorrected,” the equilibration solutions contained only Pb(NO_3_)_2_ in pure water, such that the ionic strength and initial pH of these solutions varies with the different Pb^2+^ concentrations tested (4.75 < pH < 5.50). Titrations were also carried out in 50 mM NaNO_3_ with varying Pb(NO_3_)_2_ concentrations with all solutions in a given titration adjusted to the same initial pH value (Figures 2(c) and 2(d), Tables 1 and 2). Solutions were set to an initial pH of 5.50, 4.75, or 4.00, values which span the initial pH range of the uncorrected titrations and extend to pH 4.00 on the acidic side, a value that is commonly tested in lead-binding experiments [30, 31]. Seven of the eight titrations are extremely good fits (*R* ^2^ ≥ 0.98) for the Langmuir surface-binding model [38] (see linearized Langmuir isotherm, Figure 2(b), and Table 1). In the pH uncorrected titrations (Figures 2(a) and 2(b) and Table 1), the synthetic-eumelanin-coated discs display a Langmuir affinity (*K*_*L*_) of 1.74 · 10^4^ M^−1^ and a capacity of 138 *μ*g Pb^2+^ bound per disc. Amberlite IR-120, a commercial ion-exchange resin, has a similar affinity for lead ions (*K*_*L*_= 1.2 · 10^4^) [30]. The hair-extract-coated discs display a ~2-fold-lower Langmuir affinity of 7.58·10^3^ M^−1^ and a slightly lower capacity of 126 *μ*g Pb^2+^ bound per disc.

The effect of varying the initial pH of the equilibration solutions in the tested range is relatively modest (Figures 2(c) and 2(d) and Tables 1 and 2). The affinity and capacity of the synthetic-eumelanin-coated discs in the pH 4.00 titration are 63% and 86%, respectively, of these values in the pH 5.50 titration. For the hair extract coating in the pH 4.00 titration, the affinity and capacity are 45% and 80%, respectively, of these values in the pH 5.50 titration.  Table 2 shows the percentage of Pb^2+^ bound at equilibrium for each point of these titrations; variation with pH is particularly apparent for the hair-extract-coated discs. At initial concentrations up to 30.2 *μ*M for the pH 4.75 and 5.50 titrations, or 15.1 *μ*M for the pH 4.00 titration, the synthetic eumelanin coating bound >95% of the Pb^2+^ originally in solution. Recently, Chen et al. reported that melanin extracted from squid ink is able to bind 95% of the Pb^2+^ initially present in solutions of 500 *μ*M to 2.0 mM [18]. However, it is difficult to directly compare these results to the data presented in Table 2 because of large differences in the amounts of material used (200 mg versus 1.5–3.0 mg coating per disc). Au and Potts compared the binding efficiency of synthetic eumelanin and melanin extracted from three different regions of the bovine eye (all as free suspensions), for a variety of metal ions at the same initial concentration (1.67 mM) [36]. Pb^2+^ was one of the metal ions with the highest percent bound, and the synthetic eumelanin sample had intermediate efficiency between the different natural samples; melanin extracted from the choroid was the strongest binder [36]. Here, melanin extracted from human hair is less efficient than the synthetic coating (Table 2). An enzymatic extraction procedure [27] and/or alternate deposition conditions may allow us to utilize the renewable resource of human hair for more efficient lead-binding melanin-based coatings than this first generation.

The Langmuir affinity constants (Table 1) for the hair-extract- and synthetic-coated discs are within the range of values of affinity constants (10^3^ to 10^7^ M^−1^) obtained by a variety of methods for different metal ions with synthetic and natural melanin samples [7, 38]. Earlier reports have suggested that natural and synthetic eumelanin samples have a high affinity for Pb^2+^ compared to other metal ions [35, 36], but affinity constants were not established in these studies, and until recently [18], current studies have generally not included lead. Given the known heterogeneity of both the natural and synthetic samples, the Langmuir affinity values reported here presumably represent an aggregate affinity rather than a microscopic per site affinity. On a molecular level, the catechol-like oxygens, carboxylic acids, the indole nitrogens, and the indole *π* electron density all potentially play some role in binding. In a recent study using melanin from squid ink, Chen et al. saw evidence of involvement of both the catechol and carboxylate functionalities in lead binding [18]. Affinity constants were not determined in that study but it was found that lead binding was more resistant than cadmium binding to competition with salts although this could be due differing binding modes as well as differing affinities [18].

Although the PVDF support shows little to no affinity for lead ions on its own, the structure of the support likely influences both the number and distribution of available binding sites compared to other presentations. Nevertheless, for comparison with other systems we considered binding as a function of the mass of the coating. At the maximum capacities, the synthetic eumelanin coating binds ~9% of its mass in Pb^2+^ (138 *μ*g/1.5 mg), while the hair extract coating binds ~4.5% of its mass in Pb^2+^ (139 *μ*g/3 mg). These values, which equate to 0.44 mmol (Pb^2+^)/g (coating) and 0.22 mmol/g for the synthetic eumelanin and hair extract, respectively, should be considered approximate, because of uncertainty in the average mass of the coating. Nevertheless, they are within the range of reported capacities for a variety of natural and synthetic melanin samples with different metal ions [7] and are at the higher end of heavy metal capacities for a variety of biosorbents [32]. Melanin extracted from squid ink was able to bind 13.5% of its mass in Pb^2+^ at capacity (0.65 mmol/g) [18].

To begin to assess the selectivity of melanin-based coatings for Pb^2+^ and probe the generality of the binding assay, titrations of the nitrate salts of copper (Cu^2+^), cadmium (Cd^2+^), and zinc (Zn^2+^) were performed with synthetic-eumelanin-coated discs (Figure 3 and Table 3). Ten concentrations of each metal ion in 50 mM NaNO_3 _ (aq.), initial pH adjusted to pH 4.75, were used, for comparison with the Pb^2+^ results. All three titrations were extremely good fits (*R* ^2^ ≥ 0.99) for the Langmuir model yielding *K*_*L*_ values of 1.41 · 10^4^ M^−1^ for Cu^2+^, 9.70 · 10^3^ M^−1^ for Cd^2+^, and 6.44 · 10^3^ M^−1^ for Zn^2+^. Thus, under the conditions tested, the Langmuir affinities for Zn^2+^, Cd^2+^, and Cu^2+^ were 35%, 53%, and 77%, respectively, of the Langmuir affinity for Pb^2+^. In terms of capacity, on the basis of mass bound, the discs have significantly higher capacity for lead than the other ions tested, but this is partially the result of the mass of lead itself; on per mole basis, the capacities for Zn^2+^ (383 nmol/disc, ~0.26 mmol/g coating) and Cd^2+^ (443 nmol/disc, ~0.30 mmol/g coating) are below the capacity for Pb^2+^ (629 nmol/disc, ~0.42 mmol/g coating), while the capacity for Cu^2+^ (771 nmol/disc, ~0.51 mmol/g coating) is higher. Chen et al. found that melanin from squid ink had a higher molar capacity for Cd^2+^ (0.93 mmol/g) than for Pb^2+^ (0.65 mmol/g) [18]. Copper and zinc are among the most studied metal ions for interactions with natural and synthetic melanin samples [7, 20, 33, 39]. In general Cu^2+^ is found to be the stronger binder, while the capacity for Zn^2+^ can be taken as a reflection of the DHICA content of the melanin sample [7]. For example, *Sepia *eumelanin has a higher capacity for Zn^2+^ (~1.5 mmol/g) than for Cu^2+^ (1.1 mmol/g) [7], whereas the lower capacity for zinc ions here likely reflects the lower DHICA : DHI ratio in L-dopa/tyrosinase synthetic eumelanin [34]. The generally lower capacities displayed by the synthetic-eumelanin-coating compared to natural melanin samples for the four metal ions tested likely reflect differences in both the composition of the synthetic eumelanin and its presentation on PVDF. 

A study by Buszman et al. comparing the binding of metal ions including Cu^2+^, Cd^2+^, and Zn^2+^ (but Pb^2+^) to a suspension of synthetic eumelanin generated by the autoxidation of L-dopa (i.e., a different method than used here) was recently reanalyzed [38, 40]. Fitting the data to several different models Bridelli and Crippa found the affinity order Zn^2+^< Cd^2+^ < Cu^2+^ [38], which is the same order as observed here. In their study with bovine eye melanin and synthetic eumelanin (produced by the L-dopa/tyrosinase method) Potts and Au found that Zn^2+^ had lower binding efficiency than Pb^2+^ and Cu^2+^, which had had similar binding efficiency (Cd^2+^ was not tested) [36]. Using a competitive binding assay against the nitrogen di-cation paraquat, Larsson and Tjalve found that Pb^2+^ was slightly more competitive than Cu^2+^ and that Pb^2+^, Cu^2+^, La^3+^, and Gd^3+^ were the most competitive of eighteen metal ions tested for bovine eye melanin (however, Zn^2+^ and Cd^2+^ were not tested) [35]. These authors tested a more limited subset of metal ions with synthetic eumelanin produced by the L-dopa/tyrosinase method; Pb^2+^ was again slightly less competitive than La^3+^ and more competitive than other metal ions tested (Cu^2+^ was not tested with the synthetic sample) [35]. Overall, the results for the synthetic eumelanin coated discs with the four metal ions tested here (Table 2) are consistent with affinity trends in the literature and add to the earlier findings that eumelanin binds Pb^2+^ strongly compared to other metal ions, with affinity similar to (or, under the conditions tested here, slightly higher than) its affinity for Cu^2+^. In this context, it is interesting to note that, compared to the presence of these metals in the surrounding tissue, neuromelanin concentrates lead to a much greater extent than copper [13]. 

## 4. Conclusions

 Melanins have many fascinating chemical properties in addition to their roles as biological pigments, including molecular recognition properties that are potentially advantageous for water purification applications. In this study we have developed a procedure for investigating melanin-metal ion interactions on solid support, with lead as the initial target. Melanin-based coatings derived by extraction of melanin from human hair and the biomimetic tyrosinase-catalyzed oxidation of L-dopa were shown to bind Pb^2+^ ions with reasonable affinity and high capacity, with the synthetic eumelanin coating binding up to ~9% of its mass in lead. Considered by mass bound, the synthetic-eumelanin-coated discs have a much higher capacity for Pb^2+^ than for Cu^2+^, Cd^2+^, or Zn^2+^; however, on a per mole basis the capacity for Cu^2+^ is slightly higher than for Pb^2+^. The affinity of the synthetic eumelanin coating for Pb^2+^ is slightly higher than Cu^2+^ and higher than for Zn^2+^ and Cd^2+^. Various additives can be used to alter the composition of synthetic eumelanin polymerizations, such that the binding properties of the resulting coatings can be varied in a combinatorial fashion, potentially yielding materials with greater selectivity for lead. We are investigating other methods of isolation/deposition of natural melanin from human hair (including different color samples for varied pheomelanin content), as well as second generation synthetic coatings. Results bearing on melanin biochemistry, heavy metal toxicology, and melanin-based water purification efforts will be disclosed in due course.

## Figures and Tables

**Table 1:** Lead-binding parameters.

	Synthetic-eumelanin-coated discs	
pH^a^	*K*_*L*_ (M^−1^)^b^	Capacity *μ*g/disc^b^
Uncorrected	1.74 (±0.12) · 10^4^	137.8 ± 10.1
5.50	2.21 (±0.22) · 10^4^	133.0 ± 4.2
4.75	1.83 (±0.02) · 10^4^	130.3 ± 5.5
4.00	1.40 (±0.15) · 10^4^	119.1 ± 6.5

	Hair-extract-coated discs	
pH^a^	*K*_*L*_ (M^−1^)^b^	Capacity *μ*g/disc^b^

Uncorrected	7.58 (±0.39) · 10^3^	126.1 ± 9.5
5.50	7.04 (±0.56) · 10^3^	139.1 ± 14.7
4.75	6.74 (±1.04) · 10^3^	115.6 ± 5.4
4.00^c^	3.40 (±0.20) · 10^3^	110.5 ± 9.3

^a^Uncorrected titrations are Pb(NO_3_)_2 _ in H_2_O, other titrations are Pb(NO_3_)_2_ in 50 mM NaNO_3_ with the initial pH adjusted as shown.

^b^Values calculated from linearized Langmuir isotherms from at least three independent titrations with different discs; standard deviations shown.

^c^Linearized Langmuir isotherm *R* ^2^ ≥ 0.96, all others *R* ^2^ ≥ 0.98.

**Table 2:** Percentage of Pb^2+^ bound at equilibrium.

Initial [Pb^2+^]	Synthetic eumelanin^a^			Hair extract^a^		
	pH 5.50	pH 4.75	pH 4.00	pH 5.50	pH 4.75	pH 4.00
15.1 *μ*M	>95%	>95%	>95%	82.2%	68.2%	42.6%
30.2 *μ*M	>95%	>95%	73.3%	58.6%	49.7%	29.4%
60.4 *μ*M	68.8%	68.7%	52.6%	41.2%	36.1%	22.5%
105.7 *μ*M	49.8%	46.7%	38.7%	32.1%	26.7%	16.7%
151.0 *μ*M	39.3%	36.8%	29.4%	26.9%	21.0%	14.9%
302.0 *μ*M	22.4%	21.0%	18.3%	16.1%	14.1%	10.0%
604.0 *μ*M	12.4%	11.8%	10.6%	10.8%	8.8%	6.3%
1057.0 *μ*M	8.0%	7.6%	6.7%	7.2%	5.7%	4.9%
1510.0 *μ*M	5.6%	5.6%	5.2%	5.6%	4.8%	4.3%
2265.0 *μ*M	4.0%	4.0%	3.6%	4.2%	3.4%	3.2%

^a^Percentage of Pb^2+^ bound at equilibrium for each initial concentration from the titrations shown in Figures 2(c) and 2(d).

**Table 3:** Binding parameters of synthetic-eumelanin-coated discs with different metal ions.

	*K*_*L*_ (M^−1^)^a^	Capacity *μ*g/disc^a^	Capacity nmol/disc^a^	Highest % bound (initial [M^2+^])^b^
Pb^2+c^	1.83 (±0.02) · 10^4^	130.3 (±5.5)	629 (±27)	>95% (15.1–30.2 *μ*M)
Cu^2+^	1.41 (±0.33) · 10^4^	49.0 (±3.2)	771 (±51)	92.3% (21.5 *μ*M)
Cd^2+^	9.70 (±0.74) · 10^3^	49.8 (±5.0)	443 (±45)	68.9% (16.2 *μ*M)
Zn^2+^	6.44 (±0.19) · 10^3^	25.0 (±3.2)	383 (±49)	47.9% (33.6 *μ*M)

^a^Titrations in 50 mM NaNO_3_, initial pH 4.75, binding isotherms are shown in Figures 2(c) and 3. Values calculated from linearized Langmuir isotherms (*R* ^2^ ≥ 0.99) from at least three independent titrations with different discs; standard deviations shown.

^b^For each metal ion, the highest observed percentage bound at equilibrium is listed, with the initial concentration where this occurs shown in parentheses. ^c^Values for Pb^2+^ are repeated from Tables 1 and 2.
